# Gene expression analysis of alkaline phosphatase in peri-implantitis tissue

**DOI:** 10.6026/97320630019506

**Published:** 2023-04-30

**Authors:** Saumya Mehta, Subhashree Rohinikumar, Abhinav Rajendra Prabhu, Thiyaneswaran Nesappan, Vishnu Priya Veeraraghavan, Rajalakshmanan Eswaramoorthy

**Affiliations:** 1 Department of Implantology, Saveetha Dental College and Hospitals, Saveetha Institute of Medical and Technical Sciences (SIMATS), Saveetha University, Chennai 600077, India; 2 Department of Biochemistry, Saveetha Dental College and Hospitals, Saveetha Institute of Medical and Technical Sciences (SIMATS), Saveetha University, Chennai 600077, India; 3 Department of Biomaterials, Centre of Molecular Medicine and Diagnostics (COMManD), Saveetha Dental College and Hospitals, Saveetha Institute of Medical and Medical and Technical Sciences, Saveetha University, Chennai 600077, India

**Keywords:** Alkaline phosphatase, Peri-implantitis, Polymerase Chain Reaction, mineralization

## Abstract

Peri-implantitis is a condition that causes inflammation and bone loss around dental implants. Alkaline phosphatase (ALP) is a gene involved in the inflammation of the tissue. Therefore, it is of interest to evaluate the expression of the ALP gene around the peri-implantitis site. 20 samples were collected and analyzed using qRT-PCR, and statistical analysis was performed. The results showed a significant decrease in ALP expression in infected peri-implantitis tissue compared to normal tissue, indicating that ALP gene is involved in the inflammation of peri-implantitis and correlates with clinical findings.

## Background:

In a healthy situation, a certain degree of bone loss could be anticipated over time. There is a "natural, biological, continuous alveolar bone resorption taking place after the age of 30 years," [1]. While peri-implantitis is an inflammatory process of the soft tissue surrounding an implant accompanied by bone loss that exceeds normal physiological remodelling, peri-implant mucositis is described as a reversible inflammatory lesion affecting the soft tissue in the area immediately around implants [2]. Clinicians will have to deal with peri-implant complications that need to be appropriately managed because one in four patients undergoing implant therapy is likely to develop peri-implant diseases throughout the implants, with symptoms varying in severity [3]. According to experimental research, more osteoclasts have been seen in the peri-implantitis lesion [4]. A study involving biopsies from 40 periodontitis patients and 40 peri-implantitis patients revealed that the inflammatory lesions around implants were not only twice as large as those in periodontitis but also contained more plasma cells, macrophages, and neutrophils [5]. The signalling of these lesions reflects their various pathologies: The cytokine profiles of gingival crevicular fluid samples from healthy, periodontitis and peri-implantitis locations differed [6]. The enzyme alkaline phosphatase (ALP) is a membrane-bound glycoprotein that catalyses the hydrolysis of phosphate monoesters at basic pH levels. Alkaline phosphatase is classified into four isozymes based on the region of tissue expression: intestinal ALP, placental ALP, germ cell ALP, and tissue nonspecific alkaline phosphatase or liver/bone/kidney (L/B/K) ALP [7]. Studies of the phased expression of genes during osteoblastic development and growth plate cartilage calcification provide the key to understanding the role of ALP in mineralization. ALP is expressed early in development in bone and calcifying cartilage and is found on the cell surface and in matrix vesicles. The mechanisms that regulate ALP expression are complicated, a web of interlaced signalling pathways, the details of which are still being discovered. Therefore, it is of interest to evaluate the expression of the ALP gene around the peri-implantitis site.

##  Materials and Methods:

## Selection of subjects:

Approval of the study protocol and ethical clearance were obtained from the Institutional Review Board, Saveetha Dental College Hospitals, Chennai, India. This study was designed as described elsewhere [8, 9, 10, 11, 12, 13, 14, 15, 16, 17]. Adult individuals (>18 years old), male or female, who underwent implant surgery at Saveetha dental college and hospital 3-4 months prior taking the sample. A total of 20 samples were collected for the study and were distributed into either of the two groups based on tissue type:

Group 1- Normal individuals (n=10)

Group 2- Infected individuals (n=10)

Patients who met the inclusion criteria were recruited for this study after receiving approval from the institutional review board. The treatment and study design was explained to the qualified patients, and informed consent was obtained from the voluntarily participating patients.

## Inclusion and Exclusion criteria:

## Inclusion criteria:

Patients who have undergone implant placement 3-4 months prior to sample collection.

## Exclusion criteria:

Patients <18 years of age, Patients with uncontrolled diabetes, hepatic or renal failure, or other major medical disorders or transmittable diseases, such as serious cardiovascular disease or AIDS, Rheumatic fever, heart murmur, mitral valve prolapse, prosthetic heart valve, or other disorders that necessitate prophylactic antibiotic coverage prior to invasive dental operations. Patients undergoing antibiotic, anti-inflammatory, or anticoagulant therapy 2 weeks before the baseline exam, History of drug or alcohol abuse, Individuals consuming medications which can cause gingival overgrowth, Patients smoking ≥ cigarettes/day, Pregnancy or lactation that was self-reported by the patient (this criterion is due to oral tissue changes related to pregnancy and nursing which can affect interpretation of study results), Other serious acute or chronic medical or psychological diseases

## Sample collection:

After elevating a full thickness flap at the time of implant uncovery, soft tissue sample was collected using a tissue holding forceps and stored in an eppendorf tube containing saline. Normal sound soft tissue was collected from non-infected site and granulation tissue was collected from infected site. These Eppendorf tubes were labelled accordingly.

##  RNA extraction and quantification:

The tissues were homogenised in a homogenizer with TRIzol reagent (Invitrogen, Carlsbad, CA, USA), and total RNA was isolated following the manufacturer’s instructions. The quality and quantity of the RNA isolate were analysed using NanoDrop 2000 Lite spectrophotometer (Thermo Fisher Scientific, Waltham, US). The RNA samples were stored at -20°C until further analysis.

## Reverse transcription:

Total RNA from the sample is reverse transcribed by adding RNA sample in oligo (dT)18 primer (Promega, 50 µM), deoxyribonucleotide triphosphate (dNTP, 10mM each) (New England Biolabs Inc.) and nuclease-free water and incubated at 65° C for 5 mins followed by immediate cooling with a total volume of 10 µl. This 10 µl Template RNA primer mixture is added with 5x prime buffer (New England Biolabs Inc.), murine RNAse inhibitor (New England Biolabs Inc.), and Reverse Transcriptase (New England Biolabs Inc.), and nuclease-free water was used to make up to 20 µl. The reaction mixture is incubated in PCR (MiniAmp plus thermal cycler, Thermofisher) with 300 C for 10 mins, 42° C for 30 mins, 95° C for 5 mins, and final incubation at 4°C. The cDNA derived is quantified in Nanodrop lite and stored at -20°C until further analysis.

## Expression using qrt-PCR:

The cDNA obtained is subjected to expression studies using Sybr Green (Takara, Japan) for the gene ALP. GAPDH was used as housekeeping control genes. CFX96 Realtime System (Biorad) was used for the expression studies. The PCR cycling temperature is initial denaturation at 95° C for 30 secs for 1 cycle, denaturation at 95° C for 5 secs, and annealing at 60° C for 30 secs up to 40 cycles with a melt curve. The tests were performed as duplicates and the 2^-ΔΔCq method was used to calculate the expression of the genes. Figure 2 represents the primers used in the experiment.

## Statistical analysis:

The data presented are the mean of duplicate experiments + SEM. The statistical difference between the groups was calculated using Student's T-test in Microsoft Excel 365 software. P value < 0.05 was considered statistically significant (*).

## Results:

Out of 10 peri-implantitis cases which were considered for the study, 3 implants were retrieved due to loss of stability. Soft tissue recession was seen with 2 implants which were treated with soft tissue augmentation procedure.

**Figure 1:**
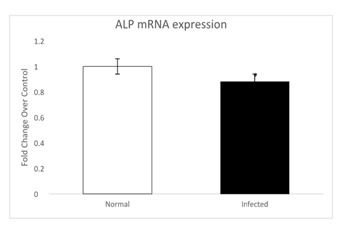
Represents the expression levels of ALP in normal and infected implant tissue sample

**Figure 2:**
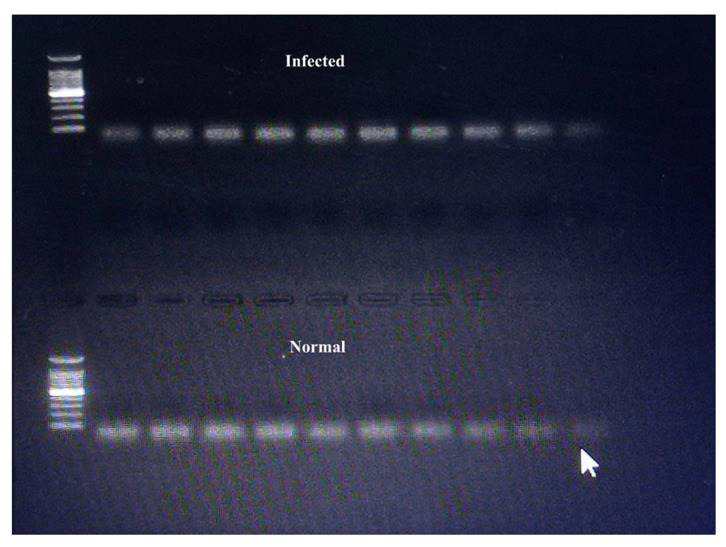
Represents the gel electrophoresis analysis of the ALP gene expression in normal and infected tissue samples

Gel electrophoresis analysis was done for both the normal and infected tissue groups and ALP m-RNA expression was found to be 1 +/- 0.2 folds for normal group as compared to infected group which was 0.8 +/- 0.1 folds as shown in Figure 1, which shows that expression of ALP gene is less in infected group as compared to the normal group.

## Discussion:

This study evaluated the gene expression of ALP in the gingival tissue of individuals with peri-implantitis compared to individuals without peri-implant disease. Overall, the results indicated that the mRNA levels of ALP were decreased in diseased tissues when compared to healthy tissues. ALP was one of the first key players in the osteogenesis process to be recognized. Subsequent research over a long period of time has confirmed the enzyme's role in normal and pathological calcification [6].ALP has been the marker of choice when examining the phenotypic or developmental maturity of mineralized tissue cells because of its centrality and convenience of the biochemical and histological assay [11]

## Conclusion:

The expression of ALP was found to be significantly lower in the infected peri-implantitis tissue when compared to normal. This concludes that the ALP gene is involved in the inflammation of peri-implantitis and it shows correlation with clinical findings. ALP gene can be used to identify the prognostic outcomes which can further help in management of peri- implantitis conditions.

## References

[1] Hugoson A (2000). Journal of Clinical Periodontology.

[2] Lindhe J (2008). Journal of Clinical Periodontology.

[3] Atieh A (2012). Journal of Periodontology.

[4] Carcuac O (2013). Clinical Oral Implants Research.

[5] Carcuac O (2014). J. Dent. Res..

[6] Gürlek O (2017). J. Periodontol..

[7] Sharma U (2014). Indian Journal of Clinical Biochemistry.

[8] Felicita AS (2022). J. Orthod..

[9] Venugopalan S (2022). J. Long Term Eff. Med. Implants.

[10] Shah KK (2022). J. Long Term Eff. Med. Implants.

[11] Shah KK (2022). J. Long Term Eff. Med. Implants.

[12] Kabilamurthi RS, Lochana GP (2022). Journal of Osseointegration.

[13] Sreenivasagan S (2021). J. Long Term Eff. Med. Implants.

[14] Sri H (2021). J. Long Term Eff. Med. Implants.

[15] Manohar J (2021). J. Long Term Eff. Med. Implants.

[16] https://www.academia.edu/58959102/International_Journal_of_Dentistry_and_Oral_Science_IJDOS.

[17] Baskran RNR (2020). J. Long Term Eff. Med. Implants.

